# Effect of UK policy on medical migration: a time series analysis of physician registration data

**DOI:** 10.1186/1478-4491-10-35

**Published:** 2012-09-25

**Authors:** Claire Blacklock, Carl Heneghan, David Mant, Alison M Ward

**Affiliations:** 1 Department of Primary Care Health Sciences, Oxford University, Radcliffe Observatory Quarter, Oxford , OX2 6GG, UK

**Keywords:** Migration, Doctors, Recruitment, United Kingdom, Africa, Policy

## Abstract

**Background:**

Economically developed countries have recruited large numbers of overseas health workers to fill domestic shortages. Recognition of the negative impact this can have on health care in developing countries led the United Kingdom Department of Health to issue a Code of Practice for National Health Service (NHS) employers in 1999 providing ethical guidance on international recruitment. Case reports suggest this guidance had limited influence in the context of other NHS policy priorities.

**Methods:**

The temporal association between trends in new professional registrations from doctors qualifying overseas and relevant United Kingdom government policy is reported. Government policy documents were identified by a literature review; further information was obtained, when appropriate, through requests made under the Freedom of Information Act. Data on new professional registration of doctors were obtained from the General Medical Council (GMC).

**Results:**

New United Kingdom professional registrations by doctors trained in Africa and south Asia more than doubled from 3105 in 2001 to 7343 in 2003, as NHS Trusts sought to achieve recruitment targets specified in the 2000 NHS Plan; this occurred despite ethical guidance to avoid active recruitment of doctors from resource-poor countries. Registration of such doctors declined subsequently, but in response to other government policy initiatives. A fall in registration of South African-trained doctors from 3206 in 2003 to 4 in 2004 followed a Memorandum of Understanding with South Africa signed in 2003. Registrations from India and Pakistan fell from a peak of 4626 in 2004 to 1169 in 2007 following changes in United Kingdom immigration law in 2005 and 2006. Since 2007, registration of new doctors trained outside the European Economic Area has remained relatively stable, but in 2010 the United Kingdom still registered 722 new doctors trained in Africa and 1207 trained in India and Pakistan.

**Conclusions:**

Ethical guidance was ineffective in preventing mass registration by doctors trained in resource-poor countries between 2001 and 2004 because of competing NHS policy priorities. Changes in United Kingdom immigration laws and bilateral agreements have subsequently reduced new registrations, but about 4000 new doctors a year continue to register who trained in Africa, Asia and less economically developed European countries.

## Background

There are 57 countries worldwide with critical shortages of health workers, many in sub-Saharan Africa; the estimated global deficit is 2.4 million doctors, nurses and midwives [1]. Economically developed nations such as the United Kingdom have the greatest density of health workers per head of population [2]. Nevertheless, along with other developed nations including the United States of America and Canada, the United Kingdom has continued to actively recruit health workers from overseas to work in locations or clinical specialties unfilled by domestic employees and to fill training posts [3-6]. Vacancies in the United Kingdom and in other developed countries are a legacy of inadequate training numbers, poor retention, particularly of nurses, and greater demand on services as a consequence of medical advancement and an ageing population [7,8].

International recruitment has sparked vehement ethical debate globally and has been blamed in part for the persisting health crisis in developing countries [3,4,7-9], with many countries losing a substantial proportion of doctors, nurses and allied health professionals. The consequences for under-resourced health systems can be substantial. Each health professional lost represents not only a considerable drain on scarce resources but also the loss of a highly trained and potentially innovative individual [8]. Migration does bring benefits: training, better working conditions, greater professional opportunities, and considerably higher salaries with associated remittances often sent home [4,10,11]. However, the danger remains that the bill for health in the developed world is paid in part by those who can least afford it. The World Health Organization recently adopted ethical guidelines for the international recruitment of health workers to try to protect struggling health systems from losing an already scarce workforce [12].

The National Health Service (NHS) employs more than 1.7 million people across England, Scotland, Wales and Northern Ireland, including more than 143 000 medical and dental staff and more than 410 000 nurses. This makes the NHS the fourth largest global employer. The NHS in England employs the majority of these staff; more than 1.4 million, serving a population of 52 million [13]. The Department of Health (DH) controls NHS activities in England. NHS services in Scotland, Wales and Northern Ireland are controlled by the respective devolved administration [13].

To be eligible to practice in the United Kingdom, either in the NHS or in private practice, a doctor must be registered with the General Medical Council (GMC). There are several limitations to using GMC registration data to estimate medical migration: registration with the GMC does not necessarily equate to current employment, or even residence in the United Kingdom; however, the data collected by the register allows an estimate of at least a presumed intention to practice in the United Kingdom from doctors who received their medical qualification overseas. In addition, registration data include the country of qualification of an individual only, not nationality or country of previous residence. There are no other national statistics providing the nationality or country of training of doctors who are actively employed in the public or private sector. Limitations of GMC data are illustrated by the discrepancy between the total number of doctors employed by the public NHS in 2005 (122 987) [13] and total number registered with the GMC in 2006 (240 328) [14]; however, we do not know the numbers of doctors employed in the private sector. Readers should consider these limitations when interpreting the findings of this paper.

At the end of 2010, 37% of the approximately 240 000 doctors registered to practice in the United Kingdom were trained overseas; almost half of these were from India or Africa [14]. This appears to be at odds with the United Kingdom government’s Code of Practice for NHS employers which counsels against recruiting medical staff from resource poor countries. Case reports suggest that this guidance may have had limited influence in the context of other NHS policy priorities [15]. We report here a time series analysis exploring the association between health policy initiatives in the NHS, including ethical guidance, and new registration of doctors who were trained overseas with the United Kingdom General Medical Council in the past decade.

## Methods

### United Kingdom policy

We identified United Kingdom policy documents relating to the migration of health workers by initially conducting a review of published articles on medical migration in the Lancet, Department of Health archives, the United Kingdom Home Office website, the Department of Health (DH) and Department for International Development (DFID) websites and World Health Organization (WHO) website. We searched for documents that were published by a United Kingdom Government department after or relating to the 2000 NHS Plan [5], published as an independent review of government policy, or published on behalf of an international organization (e.g. WHO) and relating to recruitment of health workers. We used snowballing techniques, seeking clarification by e-mail and following up reference lists. When appropriate we sought information from government departments by making requests under the Freedom of Information Act.

### General Medical Council registration data

We obtained data on the total number of new registrations with the United Kingdom GMC (including full, temporary and provisional) of doctors who obtained their primary medical qualification overseas for each year from 2000 to 2010. Each individual doctor was only counted once as a new registration, even if they later converted the type of registration (e.g. from provisional to full).

### Data analysis and presentation

We constructed figures showing time-trends in GMC registration data using Microsoft Excel software (2002 version). As the data relate to the whole of the United Kingdom, numbers are reported without estimation of sampling error. We focused on registration of doctors qualified in India, Pakistan and countries within Africa, which have both a very high disease burden and a poorly resourced health system. The key policy documents are tabulated in date order, with detail given in the supplementary tables.

## Results and discussion

### Trends in GMC registration of overseas doctors

**Figure 1:**
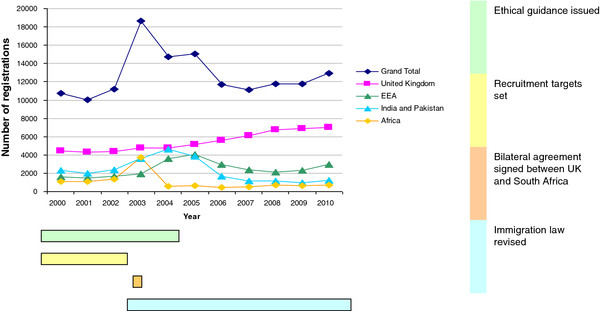
Total new United Kingdom General Medical Council registrations, 2000–2010.

**Figure 2:**
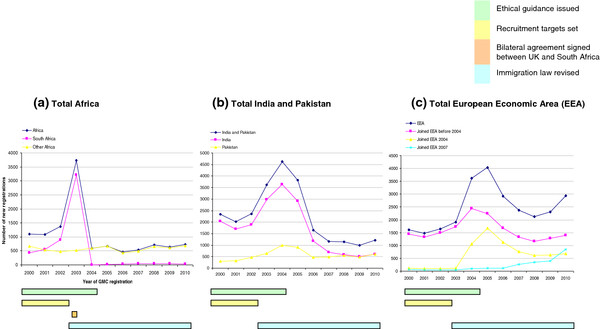
Total new United Kingdom General Medical Council registrations from Africa, India and Pakistan, and the European Economic Area.

Figure 1 shows the overall trend of GMC registrations over the 10 year period from 2000 to 2010. The most striking feature is the substantial number of new registrations by doctors trained overseas between 2003 and 2005 with only a small increase in the number of United Kingdom-trained doctors. Figure 2 shows a substantial proportion of these were by doctors trained in Africa, and India/Pakistan, although from 2004 to 2006 there was also a substantial rise in registrations from doctors trained in the European Economic Area. In 2003 there were 3728 new registrations by doctors trained in Africa, 174% higher than in 2002. In 2004 there were 4626 new registrations by doctors trained in India and Pakistan, 28% higher than in 2003 and 96% higher than in 2002. By 2006, registration of new doctors from outside the European Economic Area had fallen to a level below the rate before 2003, although in 2010 the GMC still registered 722 new doctors from Africa and 1207 new doctors from India and Pakistan.

### Policy initiatives identified

Table 1 shows the time of release of the key policy documents relevant to medical migration that we identified. They can be categorized as relating to four key issues: ethical guidance, recruitment targets, bilateral agreements between the United Kingdom and other governments, and immigration law. Further details and commentary on these policy documents are given in Additional file 1: Table S1, Additional file 2: Table S2, Additional file 3: Table S3. The temporal relationship between the timing of the policy initiatives in the four key areas and the GMC registration data is shown graphically by the coloured bars under Figures 1 and 2 and is explored in more depth below.

**Table 1:** United Kingdom policy initiatives relating to migration of doctors trained overseas

**Year**	**Title**	**Source**
Recruitment targets set		
2000 (Jul.)	The NHS Plan. A plan for investment. A plan for reform. [5]	Department of Health
2002 (Jan.)	International recruitment of consultants and General Practitioners for the NHS in England. [6]	Department of Health
Ethical guidance issued		
1999 (Nov.)	Guidance on International Nursing Recruitment. [16]	Department of Health
2001 (Oct.)	Code of Practice for NHS employers involved in the international recruitment of healthcare professionals. [17]	Department of Health
2003	List of countries published from which active recruitment was prohibited. [15]	Department of Health
2004 (Dec.)	Code of Practice for the international recruitment of healthcare professionals. [21]	Department of Health
Immigration law revised		
1999 (Nov.)	List of shortage specialties made available15	Immigration
2002/2003	Medical Act 1983 Amendment Order. [23]	Immigration
2004 (Oct.)	New member states admitted	European Union
2005	List of shortage specialties removed. [14]	Immigration
2006	Medical Act 1983 Amendment Order (draft legislation). [25]	Immigration
2006	Work permit changes. [24]	Immigration
2007 (Jan.)	New member states admitted	European Union
2008	Change in immigration law. [27]	Immigration
2010 (Nov.)	Change to immigration laws. [27]	Immigration
Bilateral agreement signed between United Kingdom and South Africa		
2003 (May)	Memorandum of Understanding between the Government of the United Kingdom of Great Britain and Northern Ireland and the Government of the Republic of South Africa on the reciprocal educational exchange of health care concepts and personnel. [22]	United Kingdom and South African Governments

### Effect of ethical guidance

The ethical code published in 1999 referred exclusively to nursing recruitment [16] but in 2001 it was updated to apply explicitly to all health workers including doctors [17]. This was updated in response to concerns about physician migration from less developed to more developed countries, in order to “promote high standards in the recruitment and employment of health professionals from abroad” [6]. In addition the code aimed to “prevent targeted recruitment from developing nations who are themselves experiencing shortages of healthcare staff” [17]. However, a specific list of 151 proscribed countries was not made available to employers until 2003 [15]. In December 2004, and after considerable criticism (see Additional file 1: Table S1), the code was updated again [18-21], now extending to recruitment of non-permanent staff, private health providers, private recruitment agencies (who were given 12 months to comply) and, included for the first time, a series of best practice benchmarks [21].

There is, however, no suggestion from the time trends in registration data that the code had an effect in reducing registrations by doctors trained in resource-poor countries. Neither the explicit inclusion of doctors in 2001, nor subsequent updates to the code were associated with any immediate reduction in new GMC registrations by doctors trained in developing countries; registration to practice in the United Kingdom increased substantially in 2003 and remained high in 2004 and 2005 (Figures 1, 2a, 2b). The stark decline in GMC registrations by doctors trained in South Africa in 2004 may have reflected ethical pressure but coincided with a Memorandum of Understanding (MoU) between the United Kingdom and South African governments [22] rather than any specific new ethical guidance to NHS employers (see below).

The explanation for the ineffectiveness of the code appears to be that it lacked any authority, relying on voluntary compliance, and failed to provide the necessary details for application by employers, as well as making no attempt to systematically monitor at a national level [6,15,18-20]. Exceptions to the code also added complication and potential ‘loopholes’: recruitment of training grades such as senior house officers (SHOs), active recruitment if a MoU existed (e.g. certain parts of India), and employment of health workers who “volunteer themselves through personal application” [17]. In addition, the code did not apply to the private sector until 2004 [21].

### Effect of recruitment targets

The NHS Plan, which was published by the United Kingdom Labour Government in 2000, set out targets for NHS employers in England to increase overall staffing numbers by an additional 7500 consultants, 2000 general practitioners, 6500 therapists and 20 000 nurses [5]. This target was rolled forward in 2002 to increase the number of new consultants and GPs further by 1000 by 2005, from the baseline in 2000 [6]. Domestic supply would clearly fall short of reaching these targets, despite plans to review salaries, improve working lives, and increase training places for medical students. The Plan, therefore, set out a strategy for active international recruitment [5]. Complementing the recruitment campaign, a list of shortage specialties had been published by the government in 1999 [15]; these were under-filled medical posts to which NHS employers could directly appoint staff from outside the EEA without providing proof of persisting vacancy. In 2002, the Government also launched an international recruitment drive to specifically increase the number of consultants and general practitioners (GPs) working in the NHS [6]. A private consultancy was employed by the United Kingdom Government to coordinate recruitment in this campaign [6].

In stark comparison to the ethical guidance to employers, the 2002 NHS recruitment drive appears highly associated with a change in GMC registrations, and is one explanation for the rise in new registrations by doctors trained in resource-poor countries which peaked in 2003–2005. Although registrations by doctors trained in these countries did begin to fall after 2005, Figure 2 also shows increasing registrations from 2005 by doctors trained in less well-resourced European countries newly admitted to the European Economic Community. The overall effect of NHS recruitment policy in England over the 10 year period is reflected by the 79 532 new overseas trained doctors who registered with the GMC between 2001 and 2010; 31% trained in India and Pakistan, 15% in Africa and 11% from other countries outside Europe and North America. The annual registration rate rose from 10 747 new registrations in 2000 to 12 929 by 2010, with a peak of 18 647 new registrations in 2003, the year after initiation of the second phase of the NHS recruitment drive, and three years after the initial NHS Plan.

### Effect of bilateral agreements between the United Kingdom and other governments

The United Kingdom Government holds MoUs with several countries regarding the recruitment of healthcare workers [18,21,22]. For some, this has been an agreement that active recruitment may take place, e.g. China, India and the Philippines [18,21]. For others, agreements have been established following concerns; the most relevant example here is the MoU with South Africa in May 2003 made in response to the South African Government requesting that active recruitment of nationals for NHS employment be curtailed [7,22]. This MoU specified that South African health personnel could, thereafter, only spend a period of time on education and practice in organizations providing United Kingdom National Health Services that was mutually agreed [22].

The effect of this MoU with South Africa on GMC registrations is shown graphically in Figure 2a. There were 3206 new registrations by doctors trained in South Africa in 2003; in 2004, the year after the MoU, there were 4 registrations. Figure 2a shows that new registrations remained very low in subsequent years. It is unclear how many of the registrations in 2003 were in anticipation of the incoming MoU, or how such anticipation might have affected registration rates in the years immediately following. Of the new 3206 registrations in 2003 by doctors trained in South Africa, 1304 (41%) of these individuals were still registered with the GMC in 2010 (GMC data). As previously stated however, GMC registration does not necessarily equate to residence or employment in the United Kingdom.

### Effect of changes in immigration law

The main changes to United Kingdom immigration law occurred after 2003. The legislation made employment of people (including doctors) from outside the European Economic Area increasingly difficult for all United Kingdom employers, by ensuring prioritization of EEA citizens for employment and introducing new work permit regulations [23,24]. Although the landmark amendment to the Medical Act, giving employment priority to EEA graduates over all other international graduates, was made in 2003, this only impacted on recruitment of overseas doctors when medical practitioners were removed from the 1999 United Kingdom shortage list of professions in 2005 [15]. Only from this time-point did employers have to prove genuine post vacancy before recruiting a non-EEA candidate. In 2006 the Medical Act was redrafted further, limiting duration of temporary registration to no more than 26 weeks in any 5-year period for visiting specialists [25]. In addition, in 2006 non-EEA doctors employed in training posts were obliged to hold work permits for the first time [24,26], and in 2008 a points-based system of immigration for highly-skilled migrants was introduced [27]. This was revised in November 2010, to allow only those highly-skilled migrants already holding job offers (known as Tier 2 migrants) to enter the United Kingdom The total number of these Tier 2 permits was also capped. Those doctors without existing job offers (known as Tier 1) who were previously able to enter the United Kingdom under the points-based system were no longer admitted [27].

These changes have been associated with a general reduction in new GMC registrations by doctors trained in non-EEA countries since 2005 (Figure 1), but particularly in new registration of doctors trained in India and Pakistan (Figure 2b, total registrations: 4626 in 2000, 1654 in 2006, 1207 in 2010). Most registrations from doctors trained in India and Pakistan between 2000 and 2006 were not full registrations, suggesting that many of these may have represented doctors in training grades who would have been particularly affected by the change in work permit policy in 2006 [24]. The impact of immigration law changes on doctors trained in Africa appears to have been less; although registration rates since 2005 are approximately half the rates seen in 2000–2002 this can be accounted for almost entirely by the effective ban on immigration of doctors from South Africa mentioned above.

The effect of the 2003 immigration legislation on new registration by doctors trained in Europe is shown clearly in Figure 2c. New registrations from the EEA peaked in 2005 at 4040. The previously mentioned increase in registration from less economically developed European countries newly joining the EAA after 2004 is also shown. The result of ten such countries joining the EU in 2004 (Malta, Cyprus, Slovenia, Estonia, Latvia, Lithuania, Poland, Czech Republic, Slovakia, Hungary) was a spike seen in new registrations in 2005. Registrations by doctors trained in Romania and Bulgaria, who joined the EU in 2007, have increased annually to 749 in 2010.

### Other factors

The United Kingdom government has referred to medical migration in several of its global policy documents (See Additional file 2: Table S2). The international community has also published several statements, including a WHO code of practice for the international recruitment of health workers (See Additional file 3: Table S3). A summary of these documents is provided in the online supplementary material but their impact on either internal policy initiatives or GMC registration rates is unclear.

## Conclusions

### Main findings

Policy decisions of a recipient country such as the United Kingdom impact significantly on the global migration of health workers. The number of doctors registering over the past decade who were trained in other countries has been closely linked to changes in DH domestic recruitment policy, immigration laws and bilateral agreements. The DH global recruitment campaign initiated in 2000 and reiterated in 2002 trumped all ethical guidance and attracted large numbers of highly trained professionals [5], in many instances from countries that could ill afford to donate. The subsequent reduction in registrations of doctors trained outside the EAA after 2005 followed changes in United Kingdom immigration policy and bilateral inter-government agreements.

The failure of the DH ethical code of practice is disappointing and reflects competing governmental priorities and lack of coordinated policy. It continued to evolve over a five-year period while employers engaged in active international recruitment [5,6,16,17,21] despite serious concerns raised about its effectiveness and authority at an early stage (See Additional file 1: Table S1) [20]. There was a failure of explicit or authoritative guidance for employers struggling to meet targets, and the code was not seen to impact significantly on registration patterns by doctors trained overseas.

The DH ethical code of practice can, however, be commended for the role it had in starting a global debate on the ethical recruitment of healthcare workers. Though the code did not effect the desired change in its beginnings, it has been a focus of considerable international discussion and a forerunner for subsequent international codes, such as the WHO 2010 code [12]. The implication and lesson for other countries and international organizations is that expressing good intention and providing guidance is insufficient; competing policy priorities must be confronted and implementation plans agreed and monitored.

### Strengths and limitations of the data

Doctors cannot work in the United Kingdom without registration with the GMC, which includes registration of country of training, making GMC data a proxy for medical migration. GMC registration data provide complete annual data for all doctors eligible to practice in the United Kingdom, as well as country of training for all registered members. The main limitations of registration, as a proxy for medical migration, are that it does not equate to employment status, or even residence in the United Kingdom. In addition, country of training does not necessarily equate to country of emigration. Subsequent emigration from the United Kingdom is also unknown.

Registration data also provide no information on push-pull influences on medical migration. Although the policy documents make clear that active overseas recruitment was an explicit United Kingdom government objective in 2003, we also recognize the importance of push factors in the migration of health workers [8]. In addition, sending country data are not included in this analysis. Although beyond the scope of this paper, we hope that further research will address these complex and diverse issues.

### The lesson from South Africa

Of particular concern was the number of registrations from South Africa in 2003, when 3206 doctors registered in just one year. It would normally have taken over 7 years for this number of doctors to register from South Africa based on the registration rate in 2000, and as long as 90 years based on the registration rate in 2010. For comparison, in the 2003/2004 registration year there were 4789 (excluding dentists) new registrations with the Health Professionals Council of South Africa Medical and Dental Professionals Board [28].

In May 2003, the United Kingdom and South African governments signed a MoU to “give cognisance to the existing commitment of the United Kingdom to ethical international workforce policies and practices……South African health personnel can spend a mutually agreed period of time on education and practice in organizations providing National Health Services” [22]. The extent to which the outflow of South African doctors to the United Kingdom in 2003 was in anticipation of this MoU is unknown; the consequence of this signed agreement was just four new GMC registrations in the following year.

### Policy implications

Movement of health workers from poor to rich countries substantially contributes to sustained global health inequalities [8]. Benefits taken by the NHS from ready-trained health workers go beyond just the high cost of training; it costs approximately £200 000 to train each United Kingdom medical student [29]. The cost to the United Kingdom of training the 3206 South African-trained doctors registering in 2003 would have been approximately £640 million, whereas a conservative estimate of the equivalent training costs for these doctors in South Africa in fees alone would be somewhere between £61 million and £100 million [30]. United Kingdom medical school places have increased since 2000 [29], but so has the annual number of new GMC registrations. Of new registrations in 2010, United Kingdom-trained doctors comprised just 54%. This casts doubt on the statement in 2008 that “the United Kingdom now trains all the healthcare professionals it needs” [31], although it is unknown how many registrants are actually in United Kingdom employment. To fulfil the WHO goal of greater self-sufficiency by developed countries in training health workers the United Kingdom needs to improve training capacity to meet demand [12].

Ethical guidance was ineffective in preventing mass registration for eligibility to practice in the United Kingdom by doctors trained in resource-poor countries between 2001 and 2004, because of competing policy priorities. Changes in United Kingdom immigration laws and bilateral agreements have subsequently reduced new registrations from resource-poor countries, but about 4000 new doctors continue to register annually who have trained in Africa, Asia and less economically developed European countries.

Voluntary codes for ethical recruitment of health workers, whether international or domestic, have limited value without coordinated policy. Guidance on ethical behaviour is obviously not enough; change in registrations resulted only from legislation and from setting and monitoring specific performance targets. In addition, improvements in workforce data collection are important and needed.

## Abbreviations

DFID: Department For International Development; DH: Department of Health; EEA: European Economic Area; GMC: General Medical Council; MoU: Memorandum of Understanding; NHS: National Health Service; OECD: Organization for Economic Cooperation and Development; PMQ: Primary Medical Qualification; RCN: Royal College of Nursing; SHO: Senior House Officer; WHO: World Health Organization.

## Competing interests

The authors declare they have no competing interests.

## Authors’ contributions

CB, CH and AW devised the study. CB extracted the data. CB and CH analysed the data. CB, CH and AW wrote the first draft of the paper. DM helped CB restructure the first draft and co-wrote the final draft submitted. All authors read and approved the final manuscript.

## Role of funding source

The Department of Primary Health Care is part of the NIHR School of Primary Care Research and is funded by the European Union to conduct research on primary care staffing in Africa as a collaborator in the HURAPRIM project.

## Supplementary Material

Additional file 1 Table S2 Summary of commentary on NHS ethical code of practice in relation to updates (abbreviations listed at end of manuscript). (PPT 46 kb) Click here for file

Additional file 2 Table 3 Summary of other related UK policy and key commentary (abbreviations listed at end of manuscript). (PPT 51 kb) Click here for file

Additional file 3 Table 4 Summary of international resolutions and codes relating to health worker migration (abbreviations listed at end of manuscript). (PPT 30 kb) Click here for file
