# Supplementary material for: Effect of UK policy on medical migration: a time series analysis of physician registration data
Source: Hum Resour Health. 2012 Sep 25;10:35. doi: 10.1186/1478-4491-10-35 (PMC3476980; doi:10.1186/1478-4491-10-35)
Supplement: Additional file 1 Table S2 — Summary of commentary on NHS ethical code of practice in relation to updates (abbreviations listed at end of manuscript). (PPT 46 kb) [file 1478-4491-10-35-S1.ppt]

## Slide 1
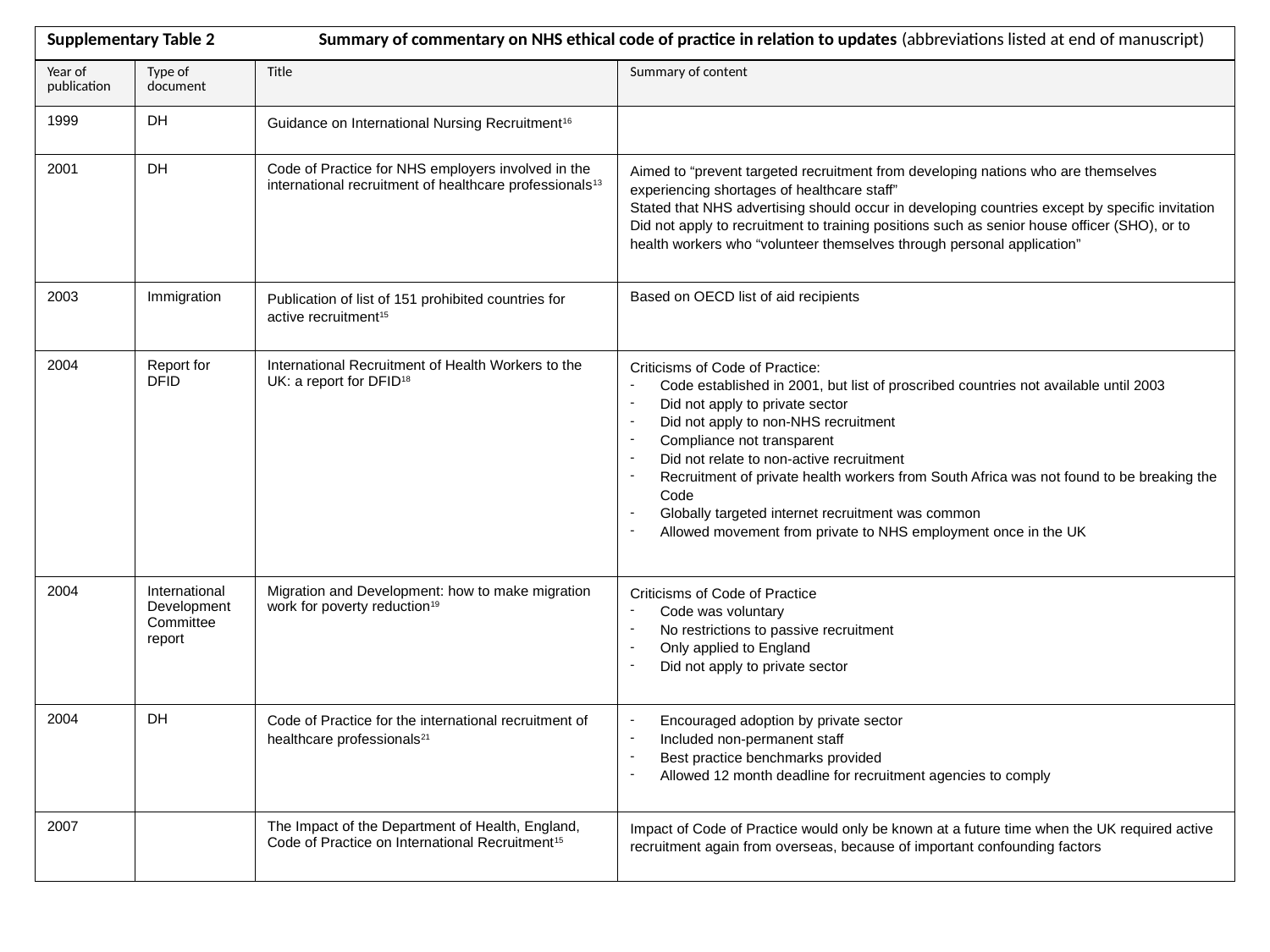

| Supplementary Table 2 Summary of commentary on NHS ethical code of practice in relation to updates (abbreviations listed at end of manuscript) | | | |
| --- | --- | --- | --- |
| Year of publication | Type of document | Title | Summary of content |
| 1999 | DH | Guidance on International Nursing Recruitment16 | |
| 2001 | DH | Code of Practice for NHS employers involved in the international recruitment of healthcare professionals13 | Aimed to “prevent targeted recruitment from developing nations who are themselves experiencing shortages of healthcare staff” Stated that NHS advertising should occur in developing countries except by specific invitation Did not apply to recruitment to training positions such as senior house officer (SHO), or to health workers who “volunteer themselves through personal application” |
| 2003 | Immigration | Publication of list of 151 prohibited countries for active recruitment15 | Based on OECD list of aid recipients |
| 2004 | Report for DFID | International Recruitment of Health Workers to the UK: a report for DFID18 | Criticisms of Code of Practice: Code established in 2001, but list of proscribed countries not available until 2003 Did not apply to private sector Did not apply to non-NHS recruitment Compliance not transparent Did not relate to non-active recruitment Recruitment of private health workers from South Africa was not found to be breaking the Code Globally targeted internet recruitment was common Allowed movement from private to NHS employment once in the UK |
| 2004 | International Development Committee report | Migration and Development: how to make migration work for poverty reduction19 | Criticisms of Code of Practice Code was voluntary No restrictions to passive recruitment Only applied to England Did not apply to private sector |
| 2004 | DH | Code of Practice for the international recruitment of healthcare professionals21 | Encouraged adoption by private sector Included non-permanent staff Best practice benchmarks provided Allowed 12 month deadline for recruitment agencies to comply |
| 2007 | | The Impact of the Department of Health, England, Code of Practice on International Recruitment15 | Impact of Code of Practice would only be known at a future time when the UK required active recruitment again from overseas, because of important confounding factors |
