# Supplementary material for: Effect of UK policy on medical migration: a time series analysis of physician registration data
Source: Hum Resour Health. 2012 Sep 25;10:35. doi: 10.1186/1478-4491-10-35 (PMC3476980; doi:10.1186/1478-4491-10-35)
Supplement: Additional file 2 Table 3 — Summary of other related UK policy and key commentary (abbreviations listed at end of manuscript). (PPT 51 kb) [file 1478-4491-10-35-S2.ppt]

## Slide 1
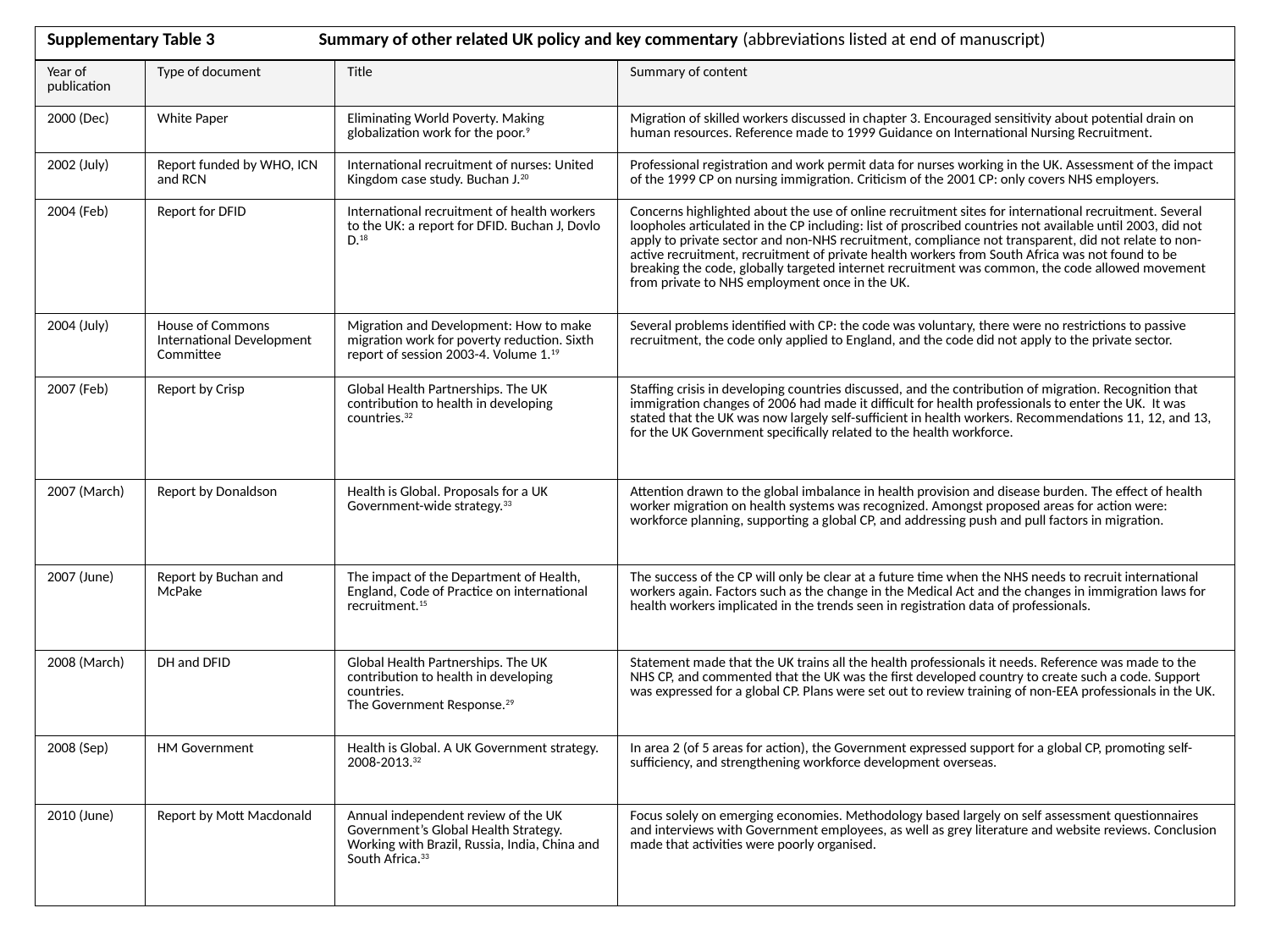

| Supplementary Table 3 Summary of other related UK policy and key commentary (abbreviations listed at end of manuscript) | | | |
| --- | --- | --- | --- |
| Year of publication | Type of document | Title | Summary of content |
| 2000 (Dec) | White Paper | Eliminating World Poverty. Making globalization work for the poor.9 | Migration of skilled workers discussed in chapter 3. Encouraged sensitivity about potential drain on human resources. Reference made to 1999 Guidance on International Nursing Recruitment. |
| 2002 (July) | Report funded by WHO, ICN and RCN | International recruitment of nurses: United Kingdom case study. Buchan J.20 | Professional registration and work permit data for nurses working in the UK. Assessment of the impact of the 1999 CP on nursing immigration. Criticism of the 2001 CP: only covers NHS employers. |
| 2004 (Feb) | Report for DFID | International recruitment of health workers to the UK: a report for DFID. Buchan J, Dovlo D.18 | Concerns highlighted about the use of online recruitment sites for international recruitment. Several loopholes articulated in the CP including: list of proscribed countries not available until 2003, did not apply to private sector and non-NHS recruitment, compliance not transparent, did not relate to non-active recruitment, recruitment of private health workers from South Africa was not found to be breaking the code, globally targeted internet recruitment was common, the code allowed movement from private to NHS employment once in the UK. |
| 2004 (July) | House of Commons International Development Committee | Migration and Development: How to make migration work for poverty reduction. Sixth report of session 2003-4. Volume 1.19 | Several problems identified with CP: the code was voluntary, there were no restrictions to passive recruitment, the code only applied to England, and the code did not apply to the private sector. |
| 2007 (Feb) | Report by Crisp | Global Health Partnerships. The UK contribution to health in developing countries.32 | Staffing crisis in developing countries discussed, and the contribution of migration. Recognition that immigration changes of 2006 had made it difficult for health professionals to enter the UK. It was stated that the UK was now largely self-sufficient in health workers. Recommendations 11, 12, and 13, for the UK Government specifically related to the health workforce. |
| 2007 (March) | Report by Donaldson | Health is Global. Proposals for a UK Government-wide strategy.33 | Attention drawn to the global imbalance in health provision and disease burden. The effect of health worker migration on health systems was recognized. Amongst proposed areas for action were: workforce planning, supporting a global CP, and addressing push and pull factors in migration. |
| 2007 (June) | Report by Buchan and McPake | The impact of the Department of Health, England, Code of Practice on international recruitment.15 | The success of the CP will only be clear at a future time when the NHS needs to recruit international workers again. Factors such as the change in the Medical Act and the changes in immigration laws for health workers implicated in the trends seen in registration data of professionals. |
| 2008 (March) | DH and DFID | Global Health Partnerships. The UK contribution to health in developing countries. The Government Response.29 | Statement made that the UK trains all the health professionals it needs. Reference was made to the NHS CP, and commented that the UK was the first developed country to create such a code. Support was expressed for a global CP. Plans were set out to review training of non-EEA professionals in the UK. |
| 2008 (Sep) | HM Government | Health is Global. A UK Government strategy. 2008-2013.32 | In area 2 (of 5 areas for action), the Government expressed support for a global CP, promoting self-sufficiency, and strengthening workforce development overseas. |
| 2010 (June) | Report by Mott Macdonald | Annual independent review of the UK Government’s Global Health Strategy. Working with Brazil, Russia, India, China and South Africa.33 | Focus solely on emerging economies. Methodology based largely on self assessment questionnaires and interviews with Government employees, as well as grey literature and website reviews. Conclusion made that activities were poorly organised. |
