# Supplementary material for: Effect of UK policy on medical migration: a time series analysis of physician registration data
Source: Hum Resour Health. 2012 Sep 25;10:35. doi: 10.1186/1478-4491-10-35 (PMC3476980; doi:10.1186/1478-4491-10-35)
Supplement: Additional file 3 Table 4 — Summary of international resolutions and codes relating to health worker migration (abbreviations listed at end of manuscript). (PPT 30 kb) [file 1478-4491-10-35-S3.ppt]

## Slide 1
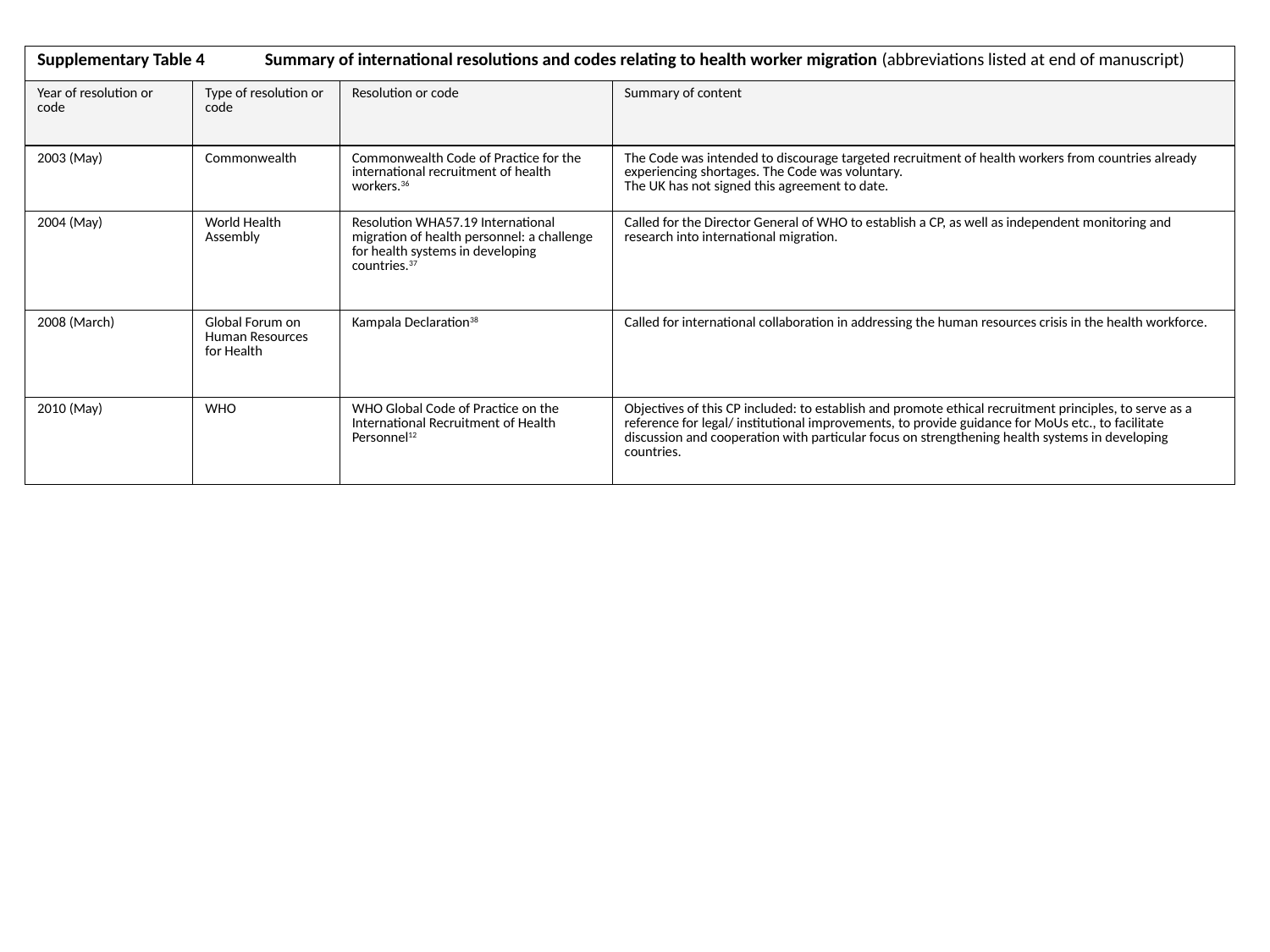

| Supplementary Table 4 Summary of international resolutions and codes relating to health worker migration (abbreviations listed at end of manuscript) | | | |
| --- | --- | --- | --- |
| Year of resolution or code | Type of resolution or code | Resolution or code | Summary of content |
| 2003 (May) | Commonwealth | Commonwealth Code of Practice for the international recruitment of health workers.36 | The Code was intended to discourage targeted recruitment of health workers from countries already experiencing shortages. The Code was voluntary. The UK has not signed this agreement to date. |
| 2004 (May) | World Health Assembly | Resolution WHA57.19 International migration of health personnel: a challenge for health systems in developing countries.37 | Called for the Director General of WHO to establish a CP, as well as independent monitoring and research into international migration. |
| 2008 (March) | Global Forum on Human Resources for Health | Kampala Declaration38 | Called for international collaboration in addressing the human resources crisis in the health workforce. |
| 2010 (May) | WHO | WHO Global Code of Practice on the International Recruitment of Health Personnel12 | Objectives of this CP included: to establish and promote ethical recruitment principles, to serve as a reference for legal/ institutional improvements, to provide guidance for MoUs etc., to facilitate discussion and cooperation with particular focus on strengthening health systems in developing countries. |
